# Spiers Memorial Lecture: Recent advances (and challenges) in supramolecular gels

**DOI:** 10.1039/d5fd00044k

**Published:** 2025-05-09

**Authors:** Haozhe Zheng, Darrin J. Pochan

**Affiliations:** a Department of Materials Science and Engineering, University of Delaware Newark DE 19716 USA pochan@udel.edu

## Abstract

Supramolecular hydrogels are physical hydrogels that are formed by non-covalent interactions such as hydrogen bonding, electrostatic attraction, hydrophobic interactions, and π–π stacking. Compared to typical, chemically cross-linked hydrogels, supramolecular networks commonly have stimuli-responsive behavior including reversibility and injectability, which are being widely studied for uses in drug delivery, tissue engineering, and wound healing. This review highlights recent developments in supramolecular network design and behavior focusing on the different possible molecular building blocks, including peptides, polysaccharides, synthetic polymers, and multicomponent systems. We further discuss self-assembly mechanisms of hydrogel formation, as well as recent advances in stimuli-responsive supramolecular hydrogels triggered by pH, temperature, and light. Advanced characterization techniques such as rheological analysis, spectroscopy, scattering methods, and electron microscopy are summarized to understand hydrogel structure, assembly pathways, and ultimate network properties. This review provides readers with an updated understanding of supramolecular hydrogels and highlights current research presented during the *Faraday Discussions* meeting on advances in supramolecular gels, promoting the rational design and development of novel materials to address complex biomedical and other technological challenges.

## Abstract 2

This review provides readers with an updated understanding of supramolecular hydrogels and highlights current research presented during the *Faraday Discussions* meeting on advances in supramolecular gels.

## Graphical abstract

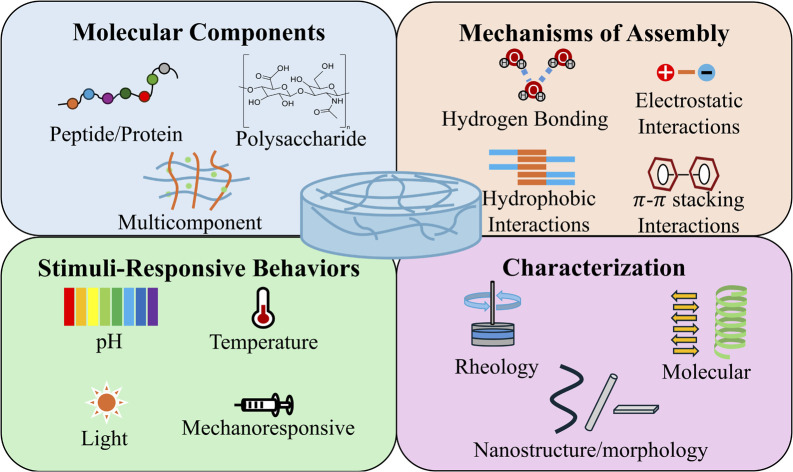

## 1. Introduction

Hydrogels are three-dimensional, hydrophilic, solid-like molecular networks that uniquely consist mostly of water and other water-soluble molecules.^1–3^ Due to their high water content and frequent cyto- and biocompatibility, hydrogels have been widely studied in wound healing, drug delivery, and soft robotics.^4–6^ Hydrogel materials can be classified into chemical hydrogels or physical hydrogels based on the driving forces and bonds used for network formation. Chemical hydrogels are fabricated by covalent interactions, with examples including radical polymerization, bioorthogonal reactions, or enzyme-catalyzed reactions for intermolecular cross-linking.^7–9^ These stable, covalent interactions impart chemical hydrogels with robust mechanical properties, as well as typically irreversible and permanent characteristics. Although an increasing number of advanced chemical hydrogels are being utilized in the biomedical field, drawbacks include a lack of application in injectable systems due to their irreversibility with respect to shear.^4,10,11^

Unlike chemical hydrogels, physical hydrogels rely on non-covalent interactions for network formation and provide unique opportunities to the materials and biomedical communities. Supramolecular hydrogels, a type of physical hydrogel, rely on molecular self-assembly for network formation through non-covalent interactions such as hydrogen bonding, hydrophobic interactions, electrostatic interactions, π–π stacking, and van der Waals forces. Since these are non-covalently cross-linked networks, supramolecular hydrogels can exhibit reversibility with respect to stimuli. For example, supramolecular networks can shift from a stiff solid to a flowing, low-viscosity material under external shear forces and recover back to the solid state when the force is removed.^12–15^ In addition, local environments can regulate the mechanical properties of supramolecular hydrogels through pH conditions,^16^ temperature,^17^ and light.^18^ These unique characteristics make injectable and adjustable supramolecular hydrogels increasingly valuable for applications in drug delivery, cell therapy, and tissue engineering.^19–22^

In this review and perspective, we summarize recent advancements in supramolecular hydrogels from approximately the last five years and emphasize new molecular building blocks, assembly mechanisms, and stimuli-responsive behaviors, as well as the advanced characterization techniques used to fundamentally understand these fascinating materials. First, we introduce the diverse molecular building blocks that researchers are using to create new supramolecular hydrogels including peptides, polysaccharides, synthetic polymers, and multicomponent systems, emphasizing the unique functionality and advantage of each. We then discuss the primary supramolecular assembly mechanisms that drive non-covalent, supramolecular assembly including hydrogen bonding, electrostatic interactions, hydrophobic interactions, and π–π stacking. Additionally, we describe recent innovations in stimuli-responsive supramolecular hydrogels triggered by pH, temperature, and light to facilitate their applications in drug delivery, tissue engineering, and biomedicine. Next, we summarize various characterization approaches, including rheological measurements, spectroscopic analyses, scattering methods, and electron microscopy to better understand the assembly mechanism and structure of hydrogels. Finally, we end with perspectives on where the field is headed and what are the areas of opportunity and need in supramolecular hydrogel research.

## 2. Molecular components of supramolecular hydrogels

The physical, chemical, and biological properties of supramolecular hydrogels are strongly influenced by the molecular components used for network formation. The components to be discussed include peptides (and some proteins), polysaccharides, synthetic polymers, and multicomponent systems, each of which contributes unique properties and potential applications across various biomedical fields.

### 2.1 Peptide supramolecular hydrogels

Peptides for peptide-based supramolecular hydrogels can be synthesized conveniently *via* solid-phase peptide synthesis (SPPS), an efficient technique that can control peptide sequence and length precisely.^23^ These hydrogels, which can be designed and synthesized from the various amino acids, exhibit excellent biocompatibility and biodegradability, making them suitable for biomedical application.^24^ Polypeptides, including both natural proteins and synthetic polypeptides, can also serve as building blocks for supramolecular hydrogel formation.^25,26^ A commonly studied polypeptide example is elastin-like polypeptides (ELP), usually composed of repeating pentapeptides (VPGXG)_*n*_ where X represents any guest amino acid residue except proline.^27^ In both peptides and polypeptides designed for supramolecular network assembly, one can take advantage of the many unique attributes of amino-acid-based biomolecules to build materials. One can use all natural amino acids for a wide range of chemical functionality. In the case of SPPS of peptides, one can also consider an essentially limitless number of non-natural chemical functionalities to include. Moreover, supramolecular hydrogels formed by peptide/polypeptide self-assembly can also exhibit well-defined secondary structures, such as α-helices and β-sheets.^28,29^ Examples abound in the literature with respect to the direct use of secondary structures for hydrogel formation. Hiew *et al.* present a short peptide hydrogel GV8 (Ac-GLYGGYGV-NH_2_) with which stiffness can be controlled by peptide concentration and ultimately reach 35.5 kPa. Initially, GV8 is in a 3_10_-helix conformation in water but gradually transitions into a β-sheet-rich fibrillar hydrogel over time.^30^ This example highlights one of the most frequently studied supramolecular mechanisms – the formation of β-sheet nano-fibrils. In the formation of β-sheet supramolecular hydrogels, peptides normally undergo secondary structural transitions from a random coil conformation to a well-defined β-sheet structure, leading to the formation of a fibril network.^31^ Restu *et al.* developed a responsive hydrogel based on a short d-peptide (d-P1) that self-assembles into β-sheet nanofibers and hydrogel networks at certain peptide concentrations through non-covalent interactions. In this example, the material is also temperature sensitive; as the temperature increases, the secondary structure of the peptide transforms into a random coil.^32^ In addition to commonly observed β-sheet structures, supramolecular hydrogels can also be formed from peptides exhibiting α-helical conformations. Typically, α-helical peptides are composed of repeating heptad sequences (abcdefg), in which positions a and d are normally hydrophobic amino acids. Two or more α-helical peptides self-assemble into coiled-coil structures driven by hydrophobic interactions among these hydrophobic residues.^33^ A recent example of helical supramolecular peptide hydrogels is from Hill *et al.* who developed a system composed of a coiled-coil pentameric protein (*Q*). This hydrogel with an α-helical coiled-coil structure can be formed at low temperatures and transitions to a liquid at 37 °C, showing reversible thermoresponsive behavior.^34^

When based on peptides/polypeptides, hydrogels can be chemically modified easily, such as with the incorporation of RGD, IKVAV or YIGSR cytoactive ligands into the peptide sequence, giving hydrogels desired biological characteristics that can promote cell adhesion and enhance cell viability.^35–37^ For example, Ishida *et al.* modified the peptide RADA16, composed of four repeating units of Arg-Ala-Asp-Ala, by replacing three continuous amino acids at four different positions with RGD. Some of these modified peptides successfully self-assembled into peptide hydrogels that exhibited enhanced cell adhesion properties.^38^ Worthington *et al.* used RGDS-MAX8 (RGDS-VKVKVKVK-V^D^PPT-KVEVKVKV-NH2) as a 3D cell culture medium for high-throughput screening.^39^ In addition to incorporation of cell adhesion peptide epitopes, peptides can be modified in other ways. For example, another important strategy in the creation of hydrogels with peptides is the design of ultrasmall molecules for supramolecular assembly, such as peptide-based low-molecular-weight gelators (LMWG). These types of molecules can self-assemble to form hydrogels, especially when functionalized with additional, non-natural functional groups to promote intermolecular assembly, such as fluorenylmethoxycarbonyl (Fmoc)-modification of dipeptides (*e.g.*, Fmoc-FF) for drug delivery.^40,41^ These small molecules are also designed for robust assembly into other targeted nanostructures. Karakaplan *et al.* silylated the dipeptide Phe–Phe and its fluorinated analogue Phe(4-F)-Phe(4-F) using 3-isocyanatopropyltriethoxysilane (ICPTES), thus making them useful for mineralization. This modification allowed the silylated dipeptides to undergo self-assembly, which led to the formation of rod-like structures for the silylated Phe–Phe contrasting with spherical structures for the silylated fluorinated analogue (https://doi.org/10.1039/D5FD00014A).

### 2.2 Polysaccharide supramolecular hydrogels

Polysaccharide-based supramolecular hydrogels, fabricated from natural resources and their derivatives *via* non-covalent interactions (*e.g.*, cellulose, alginate, chitosan, and cyclodextrins), offer low toxicity, excellent biodegradability, and bioactivity, making them excellent candidates for biomedical/biological use.^42^ For example, Wang *et al.* employed a solvent mixture technique to induce an interesting hierarchical assembly of alginate leading to the formation of a supramolecular hydrogel. Alginates initially exist as solubilized, single polymer chains in ethanol/water, but they gradually intermolecularly assemble into globules and then larger aggregates forming dendritic, snowflake-like structures. These structures further connect to form necklace-like structures, which eventually percolate to form a hydrogel with an overall nanofibrillar network. This alginate-based hydrogel is injectable, highly biocompatible, and specifically exhibits excellent hemostatic performance.^43^ At present, most polysaccharide-based supramolecular hydrogels are formed by mixing other components with polysaccharides, which we will discuss in the next section.^44–46^

### 2.3 Multicomponent supramolecular hydrogels

Multicomponent supramolecular hydrogels is, perhaps, the most quickly growing area of supramolecular gelation research. They are designed through the integration of diverse molecular building blocks, including peptides, polysaccharides, polymers, proteins, and low-molecular-weight gelators (LMWG). The diverse combination of different components enables the ultimate hydrogels to exhibit advantageous properties of each building block, thereby offering greater potential for designing multifunctional supramolecular hydrogels with desired mechanical properties, responsiveness to environmental stimuli (*e.g.* pH, temperature, and light), and/or biological performance.^47–51^ For example, Zhai *et al.* designed a multi-component supramolecular hydrogel by co-assembling Nap-GFFYGRGDHH (Pept-1) and alginate (ALG). Combining the RGD containing peptide, which promotes cell adhesion, and alginate, which enhances wound healing, this multi-component hydrogel exhibits the biological advantages of both peptides and polysaccharides.^52^ In addition, the presence of calcium ions enables the hydrogel to serve as a rapid hemostasis agent. Stereocomplexation is another example of multicomponent self-assembly systems. More *et al.* combined l-1 peptide that cannot a form hydrogel on its own with its chiral enantiomer d-1. When l-1 is mixed with bovine serum albumin (BSA), rapid self-assembly is triggered leading to b-sheet nanofibers and the formation of a stable supramolecular hydrogel. The gelation rate and the mechanical properties of the hydrogel are controlled by the ratio of BSA/l-1. However, when BSA is mixed with a different isomer, d-1, d-1 adopts a spherical conformation, resulting in a liquid state (https://doi.org/10.1039/D5FD00007F). This nicely highlights the importance peptide chirality in how one produces multicomponent, supramolecular networks (and all supramolecular networks, in general). Electrostatic interactions are commonly targeted for multicomponent molecular assembly. Some negatively charged polysaccharides, such as hyaluronic acid, cannot self-assemble into hydrogels on their own but can often form hydrogels by interacting with positively charged components through electrostatic interactions.^53^ For example, Simonson *et al.* designed a hydrogel assembled from negatively charged hyaluronic acid (HA) and positively charged peptide amphiphile poly-l-lysine (PLL).^54^

**Fig. 1:**
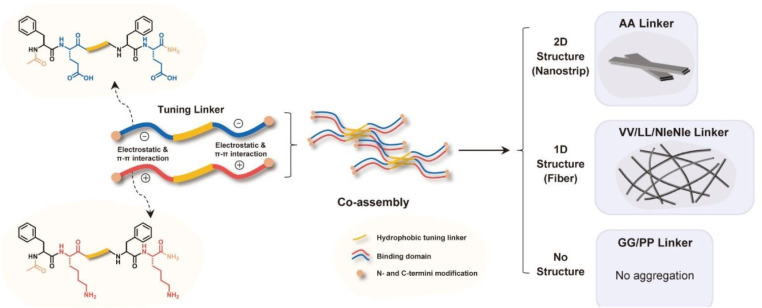
The effects of oppositely charged peptides containing different linkers on the self-assembly structure. Reproduced from https://doi.org/10.1039/D4FD00209A with permission from the Royal Society of Chemistry.

Multiple building blocks can be combined into one molecule to form multicomponent supramolecular hydrogels, including assemblies involving multiple peptides or multiple polysaccharides.^55,56^ Li *et al.* introduced six linkers (GG, AA, LL, VV, NleNle, PP) with different hydrophobic properties into the middle of the positively charged peptide (Ac-FKFK-NH_2_) and the negatively charged peptide (Ac-FEFE-NH_2_). These oppositely charged peptides with linkers are assembled into materials with different structures through electrostatic interactions, π–π interactions, and hydrophobic interactions between linkers and the phenylalanine in the two charged segments. The hydrophobic properties of the linkers can significantly affect the final morphology of the peptide assembly. In particular, the AA linker allows the peptide to eventually form a hydrogel with a two-dimensional nanobelt-like structure, while LL and VV result in the formation of a hydrogel with a one-dimensional fiber structure (Fig. 1; https://doi.org/10.1039/D4FD00209A). Ghosh *et al.* studied the effect of negatively charged aspartate or glutamate on the assembly and hydrogel formation of cationic Fmoc-Phe-diaminopropane (DAP) derivatives (Fmoc-Phe-DAP, Fmoc-3-fluorophenylalanine Fmoc-(3F)-Phe-DAP, and Fmoc-pentafluorophenylalanine Fmoc-(F_5_)-Phe-DAP). Aspartate and glutamate can affect the structure of the various cationic molecules after assembly and the formation of hydrogels. However, glutamate can induce the formation of more stable hydrogels, especially for Fmoc-3F-Phe-DAP. Whether with aspartate or glutamate, Fmoc-F_5_-Phe-DAP was the only molecule that formed similar nanofibrillar structures with both anionic additions, which may be due to the perfluorinated benzyl ring pair dominating the assembly (https://doi.org/10.1039/D4FD00198B). Amphiphilic peptides with opposite charges can co-assemble through electrostatic interactions into a two-component hydrogel with a β-sheet structure.^57^ What is clear in multicomponent systems is that the complexity of supramolecular assembly mechanisms and ultimate structure/gel properties increases significantly with only minor changes in the complexity of the constituent molecules for assembly (*e.g.*, binary mixtures). The next sections discuss the various mechanisms of assembly in more detail.

## 3. Mechanisms of supramolecular hydrogel assembly

Supramolecular hydrogels are formed through non-covalent interactions that drive network formation and stabilization. Unlike typical covalent hydrogels, supramolecular hydrogels exhibit dynamic and reversible behavior. The important mechanisms for supramolecular hydrogel formation include hydrogen bonding, electrostatic interactions, hydrophobic interactions, and π–π stacking interactions, all of which contribute to hydrogel stability, mechanical properties, and functionality. While many supramolecular systems are designed with more than one of these intermolecular interactions for assembly and gel formation, we focus on each of the interactions separately below.

### 3.1 Hydrogen bonding

**Fig. 2:**
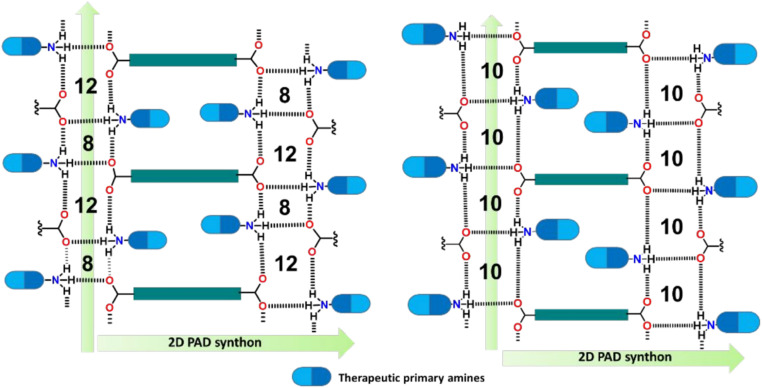
Schematic diagram of the self-assembly of PAD salts *via* hydrogen bonding. Reproduced from https://doi.org/10.1039/D4FD00154K with permission from the Royal Society of Chemistry.

Hydrogen bonding occurs when hydrogen atoms interact with electronegative atoms (N, O, and F), forming non-covalent bonds. In peptide-based hydrogels, intramolecular hydrogen bonds typically form between the amide (–NH) and carbonyl (–C=O) groups of adjacent amino acids and stabilize secondary structures such as α-helices within coiled-coil gel formers.^34^ These interactions can also be intermolecular, such as in the formation of β-sheets, leading to fibrillization and consequent hydrogel network formation.^39^ Hydrogen bonding also plays an important role in enhancing the properties of polysaccharide-based supramolecular hydrogels.^58^ While hydrogen bonding is ubiquitous in the formation of supramolecular structures with amino-acid-based molecules, one can design other non-natural small molecules for H-bonding interactions. Roy *et al.* designed what they call “vehicle-free drug delivery” (VFDD) materials for wound healing. Five dicarboxylic acids (C_4_, C_7_, C_10_, C_12_, C_14_) and four therapeutic amines (amantadine (AMN), tyramine (TRM), tryptamine (TRP), and mafenide (MAF)) were reacted to synthesize a total of 20 primary ammonium dicarboxylate (PAD) salt macromolecules. Among these, eight PAD salts can self-assemble into hydrogels through their own hydrogen bonds in methyl salicylate (MS), with most hydrogels derived from longer-chain dicarboxylic acids (Fig. 2; https://doi.org/10.1039/D4FD00154K).

Multiple hydrogen bond interactions between gelators are almost always an important part in supramolecular hydrogel assembly and the stabilization of network structure. A commonly used H-bonding motif is ureidopyrimidinone (UPy), which can form intermolecular dimers through the formation of quadruple hydrogen bonds.^59^ You *et al.* developed a highly stretchable and transparent supramolecular hydrogel based on hierarchical hydrogen bonding. The hydrogel precursors were synthesized by copolymerization of DMAA, AAc, and various molar ratios of UPy-functionalized ethyl acrylate (UPyEA). The resulting polymer network was physically cross-linked through strong UPy–UPy quadruple hydrogen bonds and weaker hydrogen bonds between DMAA and AAc. The mechanical strength and tensile strain of the hydrogels were significantly improved with increasing UPy content.^60^ Imidazolidinyl urea (IU) is another molecular tool used to provide multiple hydrogen bonds in the formation of networks. Yu *et al.* synthesized polymers containing IU to form supramolecular hydrogels, demonstrating that increasing the IU content enhances both the mechanical strength and gel–sol transition temperature of these hydrogels.^61^ Nucleic-acid-based hydrogels also take advantage of multiple H-bonds through base pairing, such as in the case of polydeoxyadenine (dA) and cyanuric acid (CA), which assemble into triple-helical fibers that further cross-link *via* base pairing to form a hydrogel network.^62^ Yang *et al.* designed a pure DNA injectable supramolecular hydrogel based on branched Y-shape DNA (BY) and self-complementary DNA linker (s-L). The two ends of s-L were designed to complement the arms of BY, resulting in the formation of a supramolecular hydrogel network.^63^ Nucleosides composed of nucleobases and cyclic pentoses assemble into supramolecular hydrogels through multiple hydrogen bonds.^64,65^ Wang *et al.* studied a nucleoside-based supramolecular hydrogel derived from 2-amino-2′-fluoro-2′-deoxyadenosine (2-FA). As a low-molecular-weight gelator (LMWG), 2-FA self-assembles through multiple hydrogen bonds to form a hydrogel with shear-thinning and self-healing properties, making it suitable for wound healing.^65^

### 3.2 Electrostatic interactions

Electrostatic interactions between oppositely charged molecules and side chains can be used for the formation, stabilization, and strengthening of supramolecular hydrogel networks.^66,67^ Criado-Gonzalez *et al.* present a system formed *via* electrostatic interactions between the amine groups of polycations and negatively charged phosphate groups of Fmoc-FFpY peptides, driving rapid self-assembly and gelation of the mixture.^68^ Dai *et al.* designed a hydrogel in which electrostatic interactions between anionic TEMPO-oxidized cellulose nanofibers (TOCNs) and cationic guar gum (CGG) played a key role in hydrogel formation.^69^ Charged components can also be added to the hydrogel network to enhance the performance of the hydrogel, such as the study of Dong *et al.* on the assembly of charged peptides that self-assemble into positively charged (K(FEFK)_2_) or negatively charged (E(FKFE)_2_) hydrogels. The incorporation of polymers with charges opposite to those of the peptide hydrogel network (poly-l-lysine or dextran) enhances the mechanical properties and stability of the original, single-component hydrogels, which is attributed to electrostatic interactions between the polymers and the network.^70^ Another example of supramolecular assembly *via* electrostatic interactions is the use of pentameric, parallel coiled-coils that form protofibrils *via* electrostatic interactions between oppositely charged N- and C-termini. Additional electrostatic interactions between protofibrils contribute to their stabilization and the consequent hydrogelation of the protein system.^71^

### 3.3 Hydrophobic interactions

Hydrophobic interactions can play a crucial role in the self-assembly and structural stability of supramolecular hydrogels, particularly in systems involving amphiphilic molecules, peptides, or synthetic polymers.^72,73^ Maki *et al.* reported the effect of hydrophobic alkyl chain length on hydrogel formation with small molecule amphiphiles; amphiphilic ureas with shorter alkyl chains (C_6_–C_9_) and lactose groups (Lac-C^*n*^) promoted hydrogel assembly.^74^ Amphiphilic peptides are well known to form β-sheet structures in conjunction with hydrophobic interactions, the combination of which facilitate their self-assembly into supramolecular hydrogels (https://doi.org/10.1039/D5FD00036J and ref. 75). Wychowaniec *et al.* studied how hydrophobic edge interactions influence the self-assembly and mechanical behavior of peptide hydrogels. Two amphiphilic peptides, F8 (FEFKFEFK) and KF8K (KFEFKFEFKK), enable self-assembly into antiparallel β-sheets through hydrophobic interactions, forming a hydrogel network composed of fibers with hydrophilic surfaces and hydrophobic cores. However, the gel properties could be fine-tuned with molecule design. Compared with F8, KF8K hides the hydrophobic residues exposed to water through the two terminal lysines. The shielded hydrophobic edge interactions inhibit the aggregation of fibers, thus forming softer and more dynamic hydrogels.^75^ Das *et al.* studied the effect of bola-amphiphilic peptides L2 (Ac-KLIIIK-NH_2_) and L5 (Ac-KIIILK-NH_2_) on self-assembly where both peptides share the same length and amino acid composition but differ in hydrophobic amino acid sequence arrangement. L2 formed nanosheets and nanoribbons while L5 self-assembled into nanotubes, demonstrating that sequence order plays a crucial role in self-assembly morphology. This structural variation results from subtle differences in β-sheet stacking (https://doi.org/10.1039/D4FD00190G). Torres-Ortega *et al.* co-assembled nanoparticles (NPs) acting as carriers of glial cell line-derived neurotrophic factor (GDNF) and hyaluronic-acid-based molecules (HA-CD/AD) into hydrogels. NPs can also act as cross-linkers through hydrophobic interactions and increase the mechanical strength of the hydrogel network.^76^ As discussed in the section on hydrogen bonding interactions, hydrophobic interactions can combine with multiple hydrogen bond interactions to drive the formation of supramolecular hydrogels.^77^ For example, Lu *et al.* developed an injectable supramolecular hydrogel based on an ABA triblock copolymer, where hydrophobic poly(methylmethacrylate) (PMMA) domains and quadruple hydrogen-bonding UPy units were combined to drive self-assembly.^78^

### 3.4 π–π stacking interactions

π–π stacking typically occurs in molecules containing aromatic groups, such as aromatic amino acids (*e.g.*, phenylalanine, tyrosine, and tryptophan) and synthetic aromatic moieties in synthetic polymers.^79,80^ The combination of peptides, particularly phenylalanine, with fluorenylmethoxycarbonyl (Fmoc) can promote the self-assembly of ultrashort peptides by facilitating π–π stacking interactions.^81–83^ Wychowaniec *et al.* investigated the influence of aromatic π-stacking interactions on the self-assembly of ultrashort, ionic complementary peptides (FEFK) and their role in hydrogel formation. They enhanced β-sheet stability by replacing phenylalanine (Phe) with phenylglycine (Phg), leading to the formation of mechanically robust supramolecular hydrogels.^84^ Zhang *et al.* combined Fmoc-FF and aromatic curcumin to form injectable drug-peptide hydrogels *via* π–π stacking interactions. The increase of curcumin loading can enhance the mechanical properties of the hydrogels due to the significant aromaticity of the added curcumin.^85^ More *et al.* developed a supramolecular hydrogel for tuberculosis therapy using graphene oxide (GO) and para-aminosalicylic acid (PAS), where π–π stacking interactions participate in the self-assembly process.^86^ Zhang *et al.* studied a supramolecular hydrogel (GBC) composed of guanosine, phenylboronic acid, and chlorogenic acid for wound healing applications. In aqueous solution, GBC forms quadruplexes that further assemble into a hydrogel network *via* π–π stacking interactions upon the addition of potassium ions.^87^

### 3.5 Other interactions

In addition to the common non-covalent interactions mentioned in the previous sections, various other interactions can also promote the formation and stabilization of supramolecular hydrogel networks. Due to its strong, directional, and reversible coordination bonds, metal–ligand coordination is used to design supramolecular hydrogels with high mechanical strength, shear thinning, and self-healing properties.^88,89^ Metal coordination plays an important role in the formation of polysaccharide-based hydrogels, especially alginate, whose carboxylate groups can coordinate with metal ions.^90^ Metal–ligand interactions can also incorporate metal ions with unique biological functions into hydrogel networks, enabling diverse biomedical applications. For instance, hydrogels can be formed through metal–ligand coordination between Ag^+^ and Fmoc-amino acids, with Ag^+^ providing antibacterial functionality to the network.^91^ In addition, host–guest interactions are also used to form supramolecular hydrogels. Macrocyclic hosts and shape-complementary guest molecules form inclusion complexes *via* non-covalent interactions.^92,93^ The cyclodextrin (CD) family is widely used as a host molecule to participate in host–guest interactions due to its truncated cone structure with a hydrophobic interior and a hydrophilic exterior.^94^ Miller *et al.* designed a shear-thinning and self-healing supramolecular hydrogel through host–guest interactions. Adamantane (Ad) as the guest or β-cyclodextrin (β-CD) as the host was grafted onto methacrylate-modified HA (MeHA) to form two types of fibers (Ad-MeHA and CD-MeHA). The reversible host–guest interaction between Ad and CD promoted the two types of fibers to form a stable supramolecular hydrogel network.^95^ Stereocomplexation involves the assembly of complementary l- and d-enantiomeric polymers or peptides through non-covalent interactions, which can be utilized to design supramolecular hydrogels.^96,97^ For example, Duti *et al.* studied the effect of stereocomplexation on the assembly and performance of supramolecular hydrogels. l- and d-pentapeptides (KYFIL) that are enantiomers of each other cannot form hydrogels on their own in water, but mixtures of l- and d-KYFIL in different proportions can form hydrogels *via* stereocomplexation. Interestingly, in PBS buffer aqueous solution, both l- and d-KYFIL independently form fibrous hydrogels with high mechanical strength, while the l/d mixture exhibits significantly reduced stiffness caused by stereocomplexation promoting the formation of plate-like structures rather than entangled nanofibers.^97^

## 4. Stimuli-responsive behaviors

Stimuli-responsive hydrogels are dynamic, adaptive materials that undergo phase transitions or structural changes in response to specific environmental cues, including pH, temperature, and light. These hydrogels are widely utilized in biomedical engineering, drug delivery, wound healing, and tissue engineering due to their ability to mimic natural biological environments. Their tunable properties allow for precise control over drug release, mechanical behavior, and self-healing functions under external stimuli.^98,99^ Supramolecular hydrogels based on non-covalent cross-linking are uniquely suited for responses to stimuli with generally more rapid responses to external stimuli than chemically cross-linked hydrogels.^72,100,101^ Examples of responsive supramolecular gel networks are discussed below.

### 4.1 pH-sensitive hydrogels

**Fig. 3:**
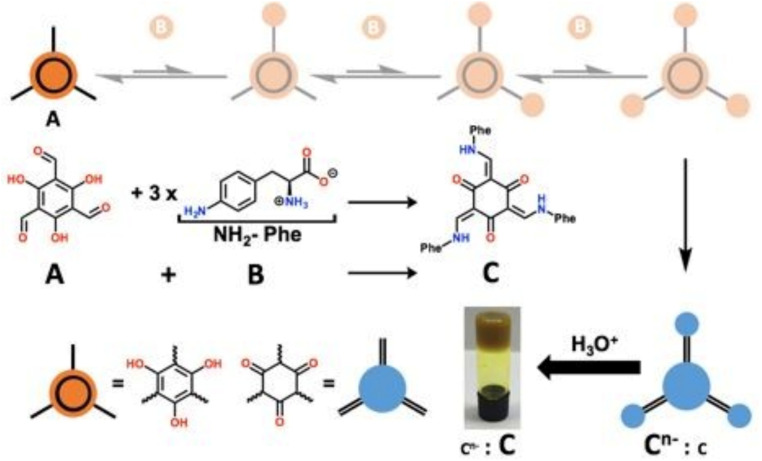
A (1,3,5-triformylglucinol) and B (4-amino-l-phenylalanine) generate C*^n^*^−^ in water at pH 8, and the protonation of C*^n^*^−^ leads to the formation of a hydrogel. Reproduced from https://doi.org/10.1039/D5FD00016E with permission from the Royal Society of Chemistry, licensed under CC BY 3.0.

pH-sensitive hydrogels undergo transitions between swelling and contracting, as well as between the sol and gel states, in response to pH changes.^102,103^ The components of these supramolecular hydrogels often contain ionizable functional groups (*e.g.*, carboxyl (–COO^−^), amine (–NH_3_^+^), and phosphate (–PO_4_^3−^) groups) that modulate hydrogel properties based on protonation or deprotonation states, especially peptides and polysaccharides.^104,105^ However, other synthetic molecules are also used. Bao *et al.* developed a pH-responsive supramolecular hydrogel based on the self-assembly of 4-arm poly(ethylene glycol)-*block*-poly(l-glutamic acid) (4a-PEG-PLG) copolymer. The hydrogel forms at low pH and transitions to a sol at high pH due to the deprotonation of the carboxyl groups of the PLG segments, enabling a pH-triggered sol–gel transformation for drug release.^106^ Foster *et al.* used imine dynamic covalent chemistry involving aldehyde and amine groups to form a gelator (C^*n*−^) from A (1,3,5-triformylglucinol) and B (4-amino-l-phenylalanine). The gelator formed hydrogels through autocatalysis autoinduction where pH plays a crucial role with high pH maintaining gelator solubility and low pH inducing protonation and triggered hydrogel formation (Fig. 3; https://doi.org/10.1039/D5FD00016E). Wang *et al.* designed a pH-responsive hydrogel by co-assembling Nap-FFKKK and curcumin. The hydrogel network formed at a pH of approximately 7.8 and disintegrated at a pH of around 5.5, triggering the controlled release of curcumin.^107^ Lee *et al.* developed a pH-responsive supramolecular hydrogel formed by the combination of coralyne (COR), which can both assist hydrogel formation and act as a therapeutic agent, and oligo-adenine strands. At neutral pH, COR and A strands form stable A–COR–A complexes that drive hydrogel formation, while at acidic pH, these structures disassemble, leading to gel dissolution and controlled COR release.^108^ The assembly mode of hydrogels can also be affected by pH, as Panja *et al.* designed a pH-responsive supramolecular hydrogel by mixing amphiphilic dipeptide with ammonium salt. At low pH, hydrogel was formed by the self-sorting of dipeptides, whereas at high pH, dipeptide co-assembly occurred with the ammonium salts thus forming mixed peptide hydrogels.^109^

### 4.2 Temperature-responsive hydrogels

Temperature-responsive hydrogels exhibit thermally induced sol–gel transitions, sometimes based on lower or upper critical solution temperatures (LCST/UCST) of the supramolecular components.^110,111^ For example, Hill *et al.* studied a UCST-type temperature-responsive hydrogel using protein nanofibers that form a gel at low temperatures and dissolve at 37 °C. The hydrogel self-assembles through nanofiber entanglement, creating a porous 3D network that enables curcumin binding, which also enhances gel mechanical stability and sustains drug release for 17–18 days.^34^ Samdin *et al.* developed an algorithm to quantify solvent-accessible hydrophobicity (SAH) and solvent-accessible charge (SAC) in peptide molecules, enabling the study of the influence of hydrophobic interactions on the temperature-dependent self-assembly of amphiphilic beta-hairpin peptide (MAX1 and its derivatives). The results show that higher SAH values promote faster fibril formation at lower temperatures (https://doi.org/10.1039/D5FD00018A). Wu *et al.* report an LCST-triggered supramolecular gelation process where hydrogel formation only occurs above the LCST transition temperature (*T*_cloud_). The hydrogel network is formed by the self-assembly of amphiphilic low-molecular-weight gelators. Below *T*_cloud_, gelators remain soluble, while above *T*_cloud_, they aggregate into macroscopic structures and assemble into a supramolecular hydrogel.^112^ Temperature-sensitive molecular components are often combined with other supramolecular components to provide thermoresponsive behavior in ultimate networks, such as poly(*N*-isopropylacrylamide) (PNIPAM) and elastin-like polypeptides (ELPs). Wang *et al.* designed a thermoresponsive supramolecular hydrogel based on PNIPAm-grafted polyurethane–urea (PUU) (PUU-g-PNIPAm) suitable for 3D printing applications. This hydrogel undergoes a hydrophilic-to-hydrophobic transition at 37 °C, at which point it also exhibits stronger mechanical properties compared to those at 20 °C.^113^ Mizuguchi *et al.* developed a supramolecular hydrogel incorporating elastin-like polypeptides (ELPs) designed to undergo a sol–gel transition around 30 °C and exhibit reversible thermoresponsive behavior.^26^

### 4.3 Light-responsive hydrogels

**Fig. 4:**
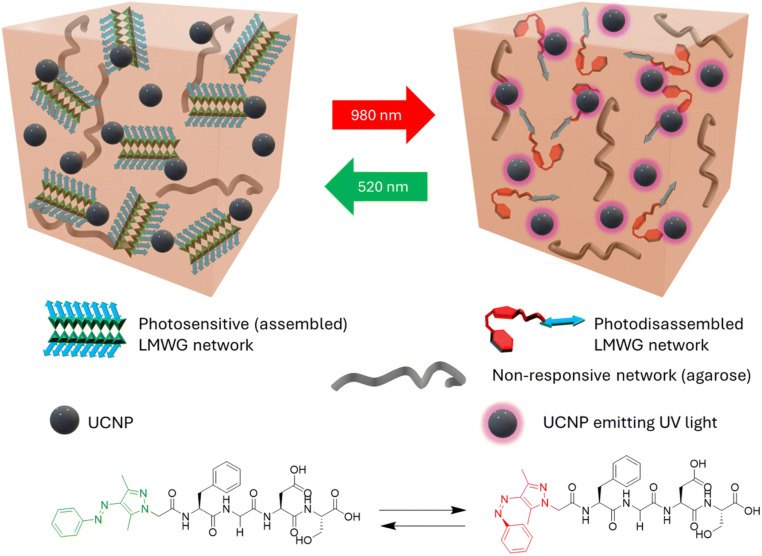
The photo-responsive three-component hydrogel contains UCNPs. When exposed to 980 nm light, the hydrogel becomes weaker. When exposed to 520 nm light, the hydrogel returns to its original intensity. Reproduced from https://doi.org/10.1039/D4FD00203B with permission from the Royal Society of Chemistry, licensed under CC BY 3.0.

Light-responsive hydrogels are hydrogels that undergo cross-linking, degradation, swelling, or shrinking upon exposure to light.^114^ In general, light-responsive hydrogels rely on the conformational photoreaction of trithiocarbonate, azobenzene, spiropyran, or coumarin derivatives by exposure to ultraviolet (UV), visible light, and near-infrared (NIR).^115–118^ In addition, Jiang *et al.* developed a light-responsive supramolecular hydrogel, poly(methacrylic acid-*co*-oligo(ethylene glycol)methacrylate) (MAA-*co*-OEGMA). A benzylimine-functionalized anthracene derivative (BIFA) was mixed into a poly(MAA-*co*-OEGMA) copolymer to form a double-cross-linked hydrogel. When the hydrogel was exposed to visible light, anthracene was able to undergo photodimerization, affecting the hydrogel network structure and causing the hydrogel to deform.^119^ Rosenbusch *et al.* combined peptide-based gelators (AAP-FGDS), agarose, and upconversion nanoparticles (UCNPs) to form a tunable three-component supramolecular hydrogel. The hydrogel undergoes a reversible gel-to-sol transition upon 980 nm NIR irradiation by UCNPs, converting NIR light into UV and triggering photoisomerization of the peptide gelator. Exposure to 520 nm visible light restores gel stiffness, allowing precise remote control over mechanical properties. Due to its unique photo-responsiveness, which only requires mild near-infrared light to regulate, this hydrogel has the potential for application in biomedicine, especially drug delivery (Fig. 4; https://doi.org/10.1039/D4FD00203B). Jeong *et al.* studied photoconductive composite hydrogels, which are formed by incorporation of three peptides (DFFD, DVVD, and DGRDKaVVD)-porphyrin dopants into a PEGDA for digital light processing (DLP) printing. Upon 415 nm light exposure, some assembled peptide-porphyrin units (DFFD and DGRDKaVVD) have higher photocurrents in salt-assembled states (https://doi.org/10.1039/D5FD00031A).

### 4.4 Mechanoresponsive hydrogels

Supramolecular hydrogels show unique mechanical responsiveness, such as shear-thinning and self-healing properties, due to their dynamic and reversible non-covalent cross-linking.^120,121^ Schneider and Pochan studied shear thinning and self-healing properties of beta-hairpin gelators. They proposed that the hydrogel network breaks under shear stress to flow and rapidly transitions back into a hydrogel network once the shear is removed. The hydrogel gradually recovers its original stiffness through the interpenetration and relaxation of fibers at the interfaces between previously fractured domains.^122^ This mechanoresponsive supramolecular hydrogel is injectable and can be widely utilized in biomedical applications such as tissue engineering, drug delivery, and wound healing.^76,123,124^ Xu *et al.* studied a thermosensitive and injectable hydrogel based on poly(*N*-acryloyl glycinamide) (PNAGA) for 3D printing. The hydrogel was softened by heating before injection to facilitate printing. After shearing ceased and a return to room temperature, the hydrogel network was reformed through hydrogen bonding interactions.^125^ The important shear thinning, self-healing properties, and consequent injectability of supramolecular hydrogels are frequently characterized through rheometry, which we will introduce in the following characterization section. While not a focus of this review, supramolecular hydrogels also can exhibit strain-stiffening characteristics where the deformation of network semi-flexible fibers causes the hydrogel to stiffen.^126^

## 5. Characterization of supramolecular hydrogels

The characterization of supramolecular hydrogels is fundamental to understanding their physical and structural properties that influence their functionality and impact in various applications. Due to the dynamic and reversible properties of non-covalent interactions, these hydrogels exhibit unique viscoelastic behavior, various self-assembly mechanisms, and distinct nanostructural features. To characterize these properties, various characterization techniques are used, ranging from rheological measurements to structural and imaging analyses. In this section, we introduce a variety of commonly used characterization techniques. While we focus on the experimental characterization of supramolecular networks, the development of new computer modeling and simulation techniques means that they are also increasingly used to understand the properties of hydrogels (https://doi.org/10.1039/D4FD00188E; https://doi.org/10.1039/D4FD00201F).

### 5.1 Rheological properties

Rheometry plays an indispensable role in the field of hydrogels. It can not only characterize the rheological properties of the hydrogel itself and the supramolecular assembly mechanisms, but rheometry can also target the changes in the hydrogel caused by its external environment, added stimuli, and internal components. Rheological characterization most typically includes measuring the storage modulus/stiffness (*G*′) and loss modulus/flow (*G*′′) through frequency-sweep measurements, strain-sweep (amplitude-sweep) measurements to observe when the network is a stiff solid *vs.* when it will fracture and flow, and time-sweep measurements to monitor the real-time assembly or disassembly of a supramolecular network.^127–129^ These three techniques have become the most important methods for characterizing the rheological properties of supramolecular hydrogels.^130–133^

**Fig. 5:**
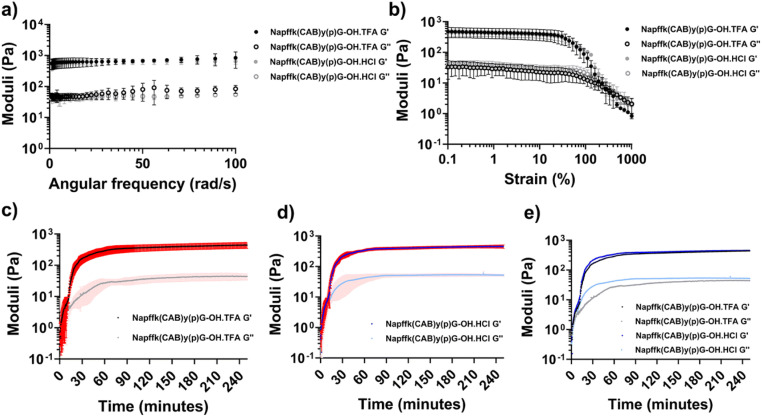
(a) Frequency sweeps and (b) strain sweeps for Napffk(CAB)yG-OH·TFA and Napffk(CAB)yGOH·HCl hydrogels. Time sweeps for (c) 2% w/v Napffk(CAB)y(p)G-OH·TFA, (d) 2% w/v Napffk(CAB)y(p)G-OH·HCl, and (e) mean values for 2% w/v Napffk(CAB)y(p)G-OH·TFA and 2% w/v Napffk(CAB)y(p)G-OH·HCl. Reproduced from https://doi.org/10.1039/D4FD00194J with permission from the Royal Society of Chemistry, licensed under CC BY 3.0.

Since the networks of supramolecular hydrogels are stabilized by non-covalent interactions, these hydrogels are usually reversible and widely utilized as injectable materials. Therefore, rheological characterization is frequently employed to measure their shear-thinning and self-healing properties.^134–136^ Yilmaz-Aykut *et al.* measured an injectable supramolecular hydrogel formed by host–guest interactions through rheological characterization. The hydrogel was subjected to strain alternately from 1% to 200% at the same frequency to characterize the shear-thinning and rehealing abilities of the hydrogel.^137^ In addition, Karga *et al.* characterized the injectable pH-responsive supramolecular hydrogel by changing the shear rate (0.01, 0.1, and 1 s^−1^).^138^ Rheometry is used to measure the changes in the mechanical properties of hydrogels under different conditions. For example, trifluoroacetate (TFA) salts are involved in the synthesis and purification of peptides and are frequently present in hydrogels made from peptide supramolecular assembly. However, TFA salts are potentially cytotoxic, and most commercial drugs are available as hydrochloride salts rather than TFA salts. Moore *et al.* studied the effects of HCl *vs.* TFA salt forms on Napffky(p)G-OH supramolecular hydrogels. The results demonstrated that gels with either salt present exhibited identical rheological properties, which was observed by frequency-, strain-, and time-sweep measurements (Fig. 5; https://doi.org/10.1039/D4FD00194J).

### 5.2 Structural and molecular characterization

**Fig. 6:**
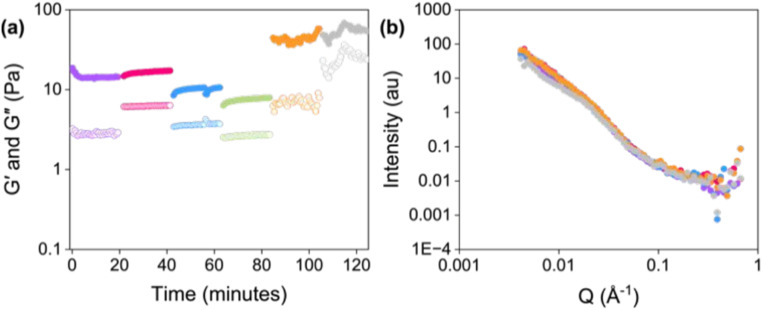
RheoSANS data of a hydrogel used for 3D printing. (a) Changes in *G*′ and *G*′′ (closed circles represent *G*′ and open circles represent *G*′′) and (b) SANS data at various shear rates (rad s^−1^). Shear rates: 0 (purple), 1 (pink), 10 (blue), 100 (green), 1000 (orange), and 2500 (red) rad s^−1^. Reproduced from https://doi.org/10.1039/D4FD00185K with permission from the Royal Society of Chemistry.

Various spectroscopic, microscopic, and scattering techniques are commonly used to investigate the self-assembly and structural characteristics of supramolecular hydrogels.^139^ Here, we introduce several widely used characterization methods that are often used in concert to understand molecular conformation through material structure and properties. Small-angle X-ray scattering (SAXS) and small-angle neutron scattering (SANS) are often used to characterize the nanostructure and larger-length-scale structure of supramolecular hydrogels.^140–142^ Edler *et al.* used SAXS analysis to explore phytantriol and monoolein in various deep eutectic solvents (DESs) and protic ionic liquids (PILs), showing that solvent composition and temperature influence observed liquid crystalline phases in the effort to understand lyotropic liquid crystal gels (https://doi.org/10.1039/D5FD00004A). Ginesi *et al.* studied the effects of 3D printing on the mechanical and structural properties of low-molecular-weight gelator (LMWG) hydrogels. SANS was used to characterize the structure of the hydrogel before and after printing. The increase in the axial ratio indicated that the fibers were more compact after printing, which was consistent with the phenomenon that the stiffness of the hydrogel increased after printing. In addition, they used RheoSANS (rheometry combined with SANS) to characterize the hydrogels at five different shear rates for 20 minutes, which can simulate the changes in the hydrogel nanostructure during the 3D printing process. The results showed that the structure of the hydrogel was an elliptical cylinder when not sheared and at low shear rates, but a combined sphere at high shear rates (Fig. 6; https://doi.org/10.1039/D4FD00185K).

Circular Dichroism (CD) can characterize the secondary structure of supramolecular hydrogels, such as the presence of α-helices and/or β-sheets.^143,144^ Meleties *et al.* explored the effect of pH on the self-assembly and gelation of a coiled-coil protein. CD spectroscopy showed negative peaks at 208 and 222 nm across pH 6, 7.4, and 10, indicating the presence of an α-helical structure at all tested conditions.^143^ Daou *et al.* developed a supramolecular hydrogel that is composed of motor tetra-acid (MTA), carboxybenzyl-protected diphenylalanine (Z-FF), and bipyridine (BPY) *via* co-assembly through hydrogen bonding. CD data show a positive peak at 190 nm and a negative peak at 206 nm, indicating the presence of a β-sheet structure. In addition, the intensities of both peaks decrease after UV exposure, indicating that UV irradiation disrupts the β-sheets, leading to the gel-to-sol transition.^145^

Fourier-transform infrared spectroscopy (FTIR) is a useful tool for investigating the driving force for supramolecular hydrogel assembly by comparing differences in spectra under various conditions.^146,147^ Alletto *et al.* used IR to characterize two-component supramolecular hydrogels composed of tripeptides (Dff-Hff and Dff-hFF). Analysis distinguished that the tripeptides were co-assembled rather than self-sorted and showed that the presence of negatively charged Asp could affect the electrostatic interactions and promote self-organization of the tripeptides (https://doi.org/10.1039/D4FD00193A). Wu *et al.* reported a supramolecular hydrogel formed by hydrogen bonding between *N*-acryloylsemicarbazide (NASC) and acrylic acid (AA). The increase and decrease of temperature promote and inhibit the dissociation of the carboxylic group, respectively, resulting in the hydroxyl peak shifting to higher and lower wavelengths in the FTIR spectrum.^148^

### 5.3 Real-space morphology characterization

Transmission electron microscopy (TEM) is a powerful imaging technique used to observe the local structure of supramolecular hydrogel networks at the nanometer scale.^149,150^ For example, Anderson *et al.* developed a sprayable supramolecular hydrogel with a collagen-binding peptide amphiphile (CBPA) for tissue adhesion and drug delivery. TEM results showed that in PBS, the hydrogel network was composed of filaments with a diameter of about 11 nm intertwined with each other.^151^ Tsutsumi *et al.* synthesized bola amphiphilic molecules that contain *N*-alkyl-2-anilino-3-chloromaleimide (AAC) (β-d-galactose (βGal)–AAC–C6–F*n* (*n* = 1–4)) with different numbers of phenylalanine (F) to study their effect on self-assembly into hydrogels. The assembled nanostructures were analyzed by TEM, where F2 and F3 formed ribbon-like and fiber structures, respectively, which may be the reason for the formation of unstable and stable hydrogels.^152^ The surface morphology and porous structure of supramolecular hydrogels can be investigated using scanning electron microscopy (SEM).^153,154^ Ding *et al.* studied a supramolecular hydrogel (G-TA) using guanosine and tannic acid for wound healing applications. SEM images showed that G-TA gel had a porous structure with a pore size of about 50 μm.^155^ An important point about SEM characterization of gels is that samples need to be processed prior to imaging in the microscope. For example, most gels need to be freeze-dried before SEM imaging. This sample preparation process typically results in the formation of ice crystals, causing structural artifacts and leading to discrepancies between the observed hydrogel morphology and its original, *in situ* structure (https://doi.org/10.1039/D4FD00204K). Additionally, while most modern SEM instruments can image non-electrically conductive materials with low accelerating voltages and low current, many gels still need to be coated with a conductive metal prior to imaging to limit charging effects. This coating can mask the *in situ* structure, particularly on the nanoscale. Overall, one must be vigilant when characterizing supramolecular gels with SEM (as well as other techniques) to limit or recognize artifacts *vs.* what is *in situ* structure and behavior.

Methods have been developed to try to see *in situ* structures while also limiting artifacts. Cryogenic Transmission Electron Microscopy and scanning electron microscopy (cryo-TEM and cryo-SEM) are used to characterize supramolecular hydrogel *in situ* structures in the hydrated state.^156,157^ Sonani *et al.* used cryo-TEM and 3D reconstruction techniques to study the atomic structure of the carbazole-modified dipeptide (CarbIF) and its self-assembly and gel formation mechanism. The results determined the atomic structure of the CarbIF hollow tube and showed that hydrophobic and hydrogen bonding forces may be the main driving forces for micelle stabilization (https://doi.org/10.1039/D4FD00181H). Li *et al.* designed a thermoresponsive supramolecular hydrogel using enzyme-assisted self-assembly (EASA). The hydrogel was formed by self-assembly of tripeptides that include phenylalanine (F) and tyrosine with a phosphate group (pY) (Fmoc-FFpY) driven by alkaline phosphatase (AP) immobilized on stellate mesoporous silica (STMS). Cryo-SEM images showed the porous structure of the hydrogel and STMS nanoparticles were observed at network cross-linking points.^158^ In order to reduce artifacts and to characterize the *in situ* structure of hydrogels, Katrantzi *et al.* report a cryo-SEM sample preparation protocol that provides artifact minimization during observation of hydrogel nanostructures. For example, the use of high-pressure freezing (HPF) can rapidly freeze samples and inhibit the formation of ice crystals. Additionally, one can focus on the edges of samples where vitrification occurred as opposed to the bulk and central regions where the thermal transfer is slower and solvent crystallization can occur, thereby reducing the impact of artifacts (https://doi.org/10.1039/D4FD00204K). This level of sophistication is what is needed in modern gel research to take the field forward.

Confocal microscopy can be used to characterize the microscale to macroscale structure of supramolecular hydrogels. The structural network of the hydrogel must be labeled with fluorescent dye and the network excited by light of a specific wavelength in order to emit fluorescence at another, characteristic wavelength for image creation.^159^ Fores *et al.* investigated the influence of hyaluronic acid (HA) on the self-assembly of enzymatic supramolecular hydrogels, and confocal microscopy revealed distinct structures of hydrogel networks depending on the presence or absence of HA.^160^ Supramolecular hydrogels are often used in the biomedical field, so confocal microscopy can also characterize the behavior of cells within hydrogels.^161^ For example, confocal microscopy can be used to analyze cell viability by characterizing the number of live (green)/dead (red) cells in hydrogels.^162^

## 6. Conclusions

Supramolecular hydrogels have become multifunctional and dynamic biomaterials and are widely used in biomedical applications such as drug delivery, tissue engineering, and wound healing. Due to their non-covalently cross-linked networks, these hydrogels exhibit stimuli-responsive behaviors, shear-thinning, and self-healing properties, leading to their great potential in advanced biomedical, 3D printing, and high-throughput screening applications. In addition, supramolecular hydrogel networks can also be easily assembled around diverse payloads, such as cells, drugs, and proteins. The careful selection and combination of molecular building blocks, including peptides and polysaccharides, and precise control of their assembly mechanisms play an important role in tuning their mechanical, structural, and biological properties. Based on their tunable stiffness, composition, and ability to encapsulate payloads, supramolecular hydrogels can mimic the extracellular matrix (ECM), making them suitable for *in vitro* testing. Advanced characterization techniques, including rheological analyses, spectroscopy, scattering methods, and electron microscopy, provide a deeper understanding of their structure and assembly mechanism. Today, increasing numbers of supramolecular hydrogels are being rationally designed to address complex biomedical challenges and enhance therapeutic outcomes. Compared to covalently cross-linked chemical hydrogels, supramolecular hydrogels assembled through non-covalent interactions generally exhibit lower mechanical strength and limited long-term stability. In some applications (*e.g.*, drug delivery, cell delivery) this may be an advantage. However, in other cases, one would like to increase the stiffness and strength of supramolecular hydrogels, such as to match the mechanical properties of much more stiff biomaterials. To address this limitation, strategies such as double-network formation^163^ and dynamic covalent interactions^164^ are being explored to improve the performance of hydrogels and make their applications more extensive. In summary, the capability of targeted design of supramolecular network physical properties (and ultimate biological or other properties) is becoming more clear with consistent study and advancements in supramolecular assembly and molecule design.

## Conflicts of interest

There are no conflicts to declare.

## Data Availability

There is no original data presented in this article. All data and information can be found in the original research articles referenced in the review.
